# Congenital Suborbital Undifferentiated Sarcoma in a Crossbred Calf

**DOI:** 10.3390/ani11020534

**Published:** 2021-02-18

**Authors:** Joana G. P. Jacinto, Marilena Bolcato, Arcangelo Gentile, Cinzia Benazzi, Luisa Vera Muscatello

**Affiliations:** Department of Veterinary Medical Sciences, University of Bologna, 40064 Ozzano Emilia (BO), Italy; joana.goncalves2@studio.unibo.it (J.G.P.J.); arcangelo.gentile@unibo.it (A.G.); cinzia.benazzi@unibo.it (C.B.); luisaver.muscatello2@unibo.it (L.V.M.)

**Keywords:** cattle, congenital tumours, undifferentiated sarcoma

## Abstract

**Simple Summary:**

Congenital tumours are rare conditions in both veterinary and human medicine. Undifferentiated sarcomas represent a recently introduced section that encompasses unclassified sarcomas that have no distinct histologic, immunohistochemical, or genetic features. The aim of this study is to describe the clinical and pathological phenotype of a 3-day-old male crossbred calf presenting a congenital suborbital mass that infiltrated the underlying muscle and bone, and suggestive of undifferentiated sarcoma. To the best of our knowledge, our report constitutes the first clinical, ultrasonographic, radiographic, endoscopic and pathological study of a congenital undifferentiated sarcoma, a condition that has been rarely reported in the veterinary literature.

**Abstract:**

Undifferentiated sarcomas are rare conditions that represent a group of unclassified sarcomas. The purpose of this study is to describe the clinical and pathological features of a calf showing a congenital infiltrating suborbital mass suggestive of undifferentiated sarcoma. The animal was referred because of respiratory distress and the presence of a right suborbital mass since birth. At ultrasonography, the mass displayed an irregular shape with multiple cavities. Radiographs revealed a diffuse, poorly defined mass with different densities overlying the bony structures of the skull. Endoscopy showed a co-involution of the mass in the right side with extension into the nasopharynx. Post-mortem examination showed a round, poorly demarcated neoplasia infiltrating the nasal turbinate and displacing the nasal septum. Histologically, the subcutis was expanded by lobules and bundles of densely cellular neoplastic spindle cells. The neoplasm infiltrated the underlying muscles, bone and the right retromandibular lymph node. The neoplastic cells had a diffuse intense cytoplasmic immunexpression to vimentin, and were negative to cytokeratin AE1/AE3, desmin, MUM1, IBA1, melan A, chromogranin and synaptophysin.

## 1. Introduction

Congenital tumours are rare conditions that arise from tissue rudiments undergoing development and are detected during pregnancy or within the first three months of life [1]. These tumours are relatively uncommon in calves, similarly to those in children [2]. Undifferentiated sarcomas are malignant tumours of mesenchymal origin and represent a recently introduced section that encompasses unclassified tumours that have no distinct histologic, immunohistochemical, or genetic features [3]. In human medicine, tumours in this category are subclassified according to the predominant morphologic patterns of round, spindle, epithelioid, or pleomorphic cells [4]. Many pleomorphic tumours are high grade and associated with poor prognosis [4].

In cattle, the only reported case of an undifferentiated sarcoma dates back to Görig in 1893 [5]. It was characterized by a multicentric form in the maxillary bone, lung, kidney, spleen and muscles of the fore and hind limbs [5].

Therefore, we aimed to typify the clinical and pathological features of a crossbred calf displaying lesions suggestive of undifferentiated sarcoma.

## 2. Materials, Methods and Results

### 2.1. Case Description and Clinical Findings

**Figure 1:**
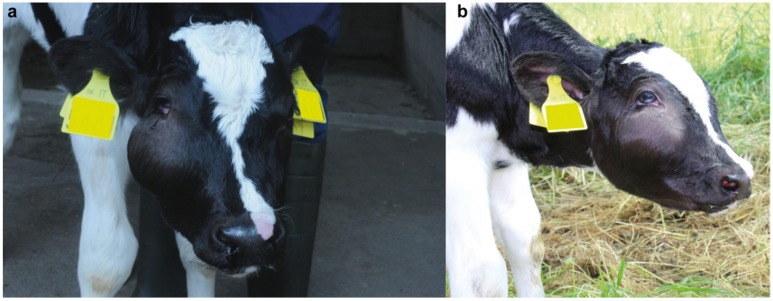
Calf showing the right sub-orbital subcutaneous mass (**a**) Frontal view of the calf. Note the presence of unilateral epistaxis and epiphora (right side). (**b**) Right lateral view of the calf. Note that the mass was delimited ventrally by the maxillary tuberosity and dorsally by the zygomatic arch compressing the lower eyelid and consequently inducing a deformation of the latest.

A 3-day-old male crossbred calf, with a body mass of 37 kg, was referred to the teaching hospital of the Department of Veterinary Medical Sciences (DIMEVET), University of Bologna, because of respiratory distress and presence of a right suborbital mass since birth. The calf was subsequently clinically examined, on which the mass, estimated to be approximately 10 cm in diameter, was recognized to be within the subcutaneous tissue (Figure 1a,b). The mass was delimited ventrally by the maxillary tuberosity and dorsally by the zygomatic arch compressing the lower eyelid. On palpation, the mass was not painful, not warm, not fluctuating, and compact in consistency, without the permanence of any fovea upon digital compression. On respiratory system examination, a semi-opened mouth breathing was detected; an evident bilateral movement of the alar cartilage, unilateral epistaxis and epiphora (right side), and absence of the air column in the right nostril were noticed. On percussion, a dull resonance at the right caudal frontal sinus and a barrel-like resonance at the level of the rostro-medial frontal sinus were perceived. On palpation of the larynx and trachea, the cough was easily determined and, on auscultation, a rattling sound was noticed. In addition, the animal revealed a tachypnoeic (45 rpm) snoring, shallow, predominantly abdominal breathing. On pulmonary auscultation wheezing was perceived in the right mid-media and mid-anterior areas of the thorax. On digestive system examination, the inspection of the oral cavity showed a remarkable deviation of the hard and soft palate. In addition, a moderate amount of fresh blood was present in the faeces (haematochezia). Clinical examination of the cardiovascular, urinary, musculoskeletal, and nervous systems showed no abnormalities.

A complete blood count (CBC) and blood chemistry profile were performed. The blood investigation of the calf showed a moderate hypoproteinaemia (5.19 g/dL) with hypoalbuminemia (2.51 g/dL) and hypogammaglobulinemia (gamma globulins = 0.64 g/dL), hypercreatinemia (7.51 mg/dL), and hyperphosphatemia (10.7 mg/dL). No abnormalities were detected in the CBC.

Blood samples from the calf were also sent for routine viral and parasitological analysis (bovine viral diarrhoea, Schmallenberg virus, bluetongue virus, Neospora spp., Toxoplasma spp.) using antigen-enzyme-linked immunosorbent assay (ELISA) and antibody-polymerase chain reaction (PCR). These analyses revealed positivity for bovine Schmallenberg virus using antigen-ELISA, and tested negative for all the remaining viral and parasitological analyses.

**Figure 2:**
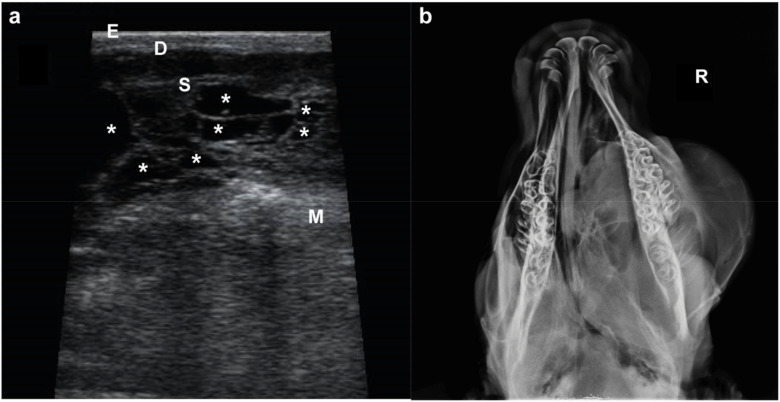
Imaging of the suborbital mass. (**a**) US image showing the three superficial layers (E, epidermis; D, dermis; S, subcutaneous tissue) overlying the malaris muscle (M) (note the bright hyperechoic appearance) and the mass with an irregular shape and multicavitary (*) (note the septated cavities filled with an anechogenic content) delimitated by S and M. (**b**) Radiography of the head in a dorso-ventral view (note that the mass extends to the left side of the hard palate). R, right side.

Ultrasound examination (US) of the infraorbital mass and radiography of the head were performed. At US the mass displayed an irregular shape with a multiple cavities (Figure 2a). Radiographs were performed to the skull in a dorsal-ventral view, and revealed a diffuse, poorly defined mass with different densities overlying the bony structures of the skull.

Moreover, endoscopy of nasal cavities and larynx was performed with a flexible endoscope. The larynx displayed a slight thickening of the medial portion of the left arytenoid in absence of laryngeal aditus reduction. The right nasal cavity, from the middle meatus and extending dorsally, was filled with a mass, characterized by smooth surface and dark colour. Endoscopy showed that the mass heavily infiltrated the nasopharynx (Video S1). On the left nasal cavity, no abnormalities were noticed.

The clinical findings were compatible with an invasive neoplasm.

The animal was euthanized at 9 days of age and was subsequently submitted to necropsy.

### 2.2. Pathological Findings

**Figure 3:**
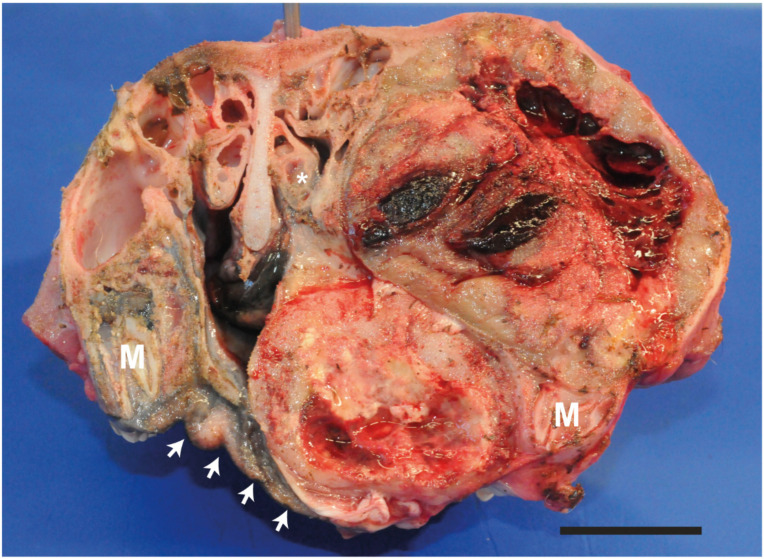
Macroscopical aspect of the suborbital congenital undifferentiated sarcoma upon transversal section at the level of the last molars (rostral aspect). Note that the neoplasm disrupts the morphology of the nasal cavities and of the palate profile (arrows) by squeezing all right nasal meatus and infiltrating the ventral conca (*). Ventrally the neoplasm displaces the palate and slightly the nasal septum to the left. The neoplasm appears multilobular, poorly demarcated, cavitated with multiple haemorrhagic and necrotic areas. M, last molar. Bar 4 cm.

Macroscopically, the mass was round, poorly demarcated, infiltrating the ventral conca and disrupting the morphology of the nasal cavities and palate (Figure 3). All right nasal meatus appeared squeezed. Ventrally, the neoplasm displaced the palate and slightly the nasal septum to the left. The neoplasm was whitish, multicavitated and with multiple haemorrhagic and necrotic areas. No macroscopically visible lesions were observed in the others organs. The mass, draining lymph nodes and internal organs were collected. The samples were formalin fixed and paraffine embedded, and routinely stained with haematoxylin and eosin for histological examination. 

**Figure 4:**
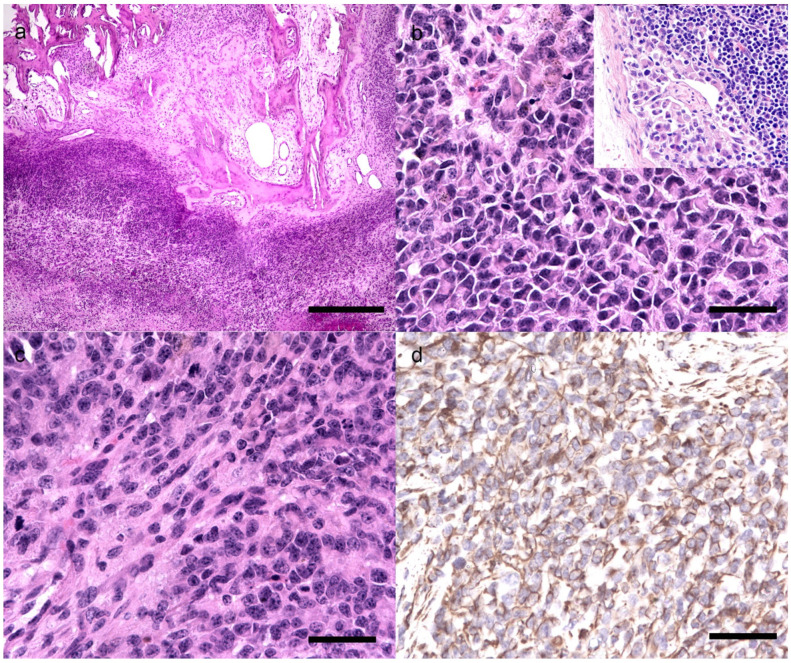
Histological and immunohistochemical features of undifferentiated sarcoma. (**a**) Solid lobules of neoplastic cells invading the bone trabeculae which have scalloped edges, HE, 40×, bar 500 micron. (**b**) Round to polygonal neoplastic cells with intermediate N/C ratio, marked anisocytosis and anisokaryosis, and numerous mitotic figures, HE, 400×, bar 100 micron; Inset: lymph node metastasis: neoplastic cells invading the subcapsular sinus. (**c**) Spindle to round neoplastic cells suggestive of highly heterogenous morphology of the neoplasm, HE, 400×, bar 100 micron (**d**) Positive immunoexpression of vimentin in the cytoplasm of neoplastic cells, 400×, bar 100 micron.

Histologically, the subcutis was expanded by the neoplasm, multilobular, densely cellular, not demarcated, unencapsulated, with infiltrative growth in the underlying skeletal muscle and bone, with lysis and resorption (Figure 4a). The neoplasm was composed of solid lobules and bundles of round to polygonal to spindle cells, embedded in a scant amount of collagenous matrix. The cells measured 15–25 micron, had distinct cell borders, an intermediate nucleus/cytoplasmic (N/C) ratio and a moderate amount of eosinophilic cytoplasm. The nuclei were oval, central to paracentral, with finely stippled chromatin and 1–3 distinct nucleoli. Anisocytosis and anisokaryosis were severe. Mitosis were 3–4 per high power field (HPF) (Figure 4b,c). In the neoplasm, there were multiple foci of abundant colliquative necrosis and haemorrhages. The right retromandibular lymph node was metastatic with infiltration of the neoplastic cells in the subcapsular sinus (Inset, Figure 4b). No metastases were detected in the internal organs. The morphological diagnosis was of an undifferentiated malignant neoplasm and immunohistochemistry (IHC) for vimentin, AE1/AE3, desmin, MUM1, IBA1, Melan A, chromogranin and synaptophysin were performed on 3-micron-thick cut sections. The neoplastic cells had a diffuse intense cytoplasmic immunexpression to vimentin (Figure 4d), and were negative to cytokeratin AE1/AE3, desmin, MUM1, IBA1, Melan A, chromogranin and synaptophysin.

The pathological and immunohistochemical findings were suggestive of undifferentiated sarcoma.

## 3. Discussion

Congenital undifferentiated sarcomas have been poorly described in veterinary medicine. To the authors’ knowledge, only one case has been so far reported in cattle and dates back to 1893 [5]. It was a multicentric form of a congenital undifferentiated sarcoma in a 3-day-old calf with a very scant description [5]. Complete histopathologic and immunohistochemical diagnosis are often not performed in calves due to lack of resources, diagnostic tools and/or low value of the affected animals, preventing the achievement of this kind of final diagnosis.

The crossbred calf presented in this study showed a congenital suborbital lesion in the subcutaneous tissue with infiltration of the underlying muscle and bone, and metastasis in the right retromandibular lymph node. The pathological and immunohistochemical results were overall suggestive of congenital round to spindle cells undifferentiated sarcoma.

A diagnosis of undifferentiated malignant neoplasm was considered and a supportive IHC was required. The negative staining to desmin, MUM1, IBA1, Melan A, chromogranin and synaptophysin excluded the diagnoses of rhabdomyosarcoma, pleomorphic plasmacytoma, histiocytic sarcoma, melanoma and malignant neuroendocrine tumour. The vimentin only positive expression suggests the mesenchymal origin of the neoplasm and therefore the diagnosis of undifferentiated sarcoma.

Rare form of spindle-cell undifferentiated sarcoma is described in adult cattle. This late-onset form of undifferentiated sarcoma may be observed in a multicentric form [6] or in the head as primary site [7], with lung and lymph node metastasis. Immunohistochemically, these forms display positivity for vimentin staining [6,7]. The aetiology of such entities remains unclear. However, for the multicentric form, intracytoplasmic virus-like particles were found in many neoplastic cells but the significance of such particles requires further investigation [6]. Interestingly, spindle-cell undifferentiated sarcomas have also been reported in cats but they are restricted to the intraocular region [8]. Pleomorphic undifferentiated sarcomas might also occur in adult cattle and similarly to humans, the primary site of the neoplasms are the limbs; usually, they are associated to metastasis in lymph nodes and the lung [9].

In human medicine, cases of congenital undifferentiated sarcomas are rare [10,11,12,13]. Some of these entities might be located in the orbital region and histologically appear as locally aggressive pleomorphic undifferentiated sarcomas with invasion of the underlying soft tissue and bone [11]. Others are located in the retroperitoneal space and histologically display small round cells showing also locally aggressive infiltration of the surrounding soft tissues and bone; these neoplasms are associated to BCOR-CCNB3 gene fusion [10]. Although congenital, the undifferentiated sarcomas reported in humans are histologically different from the case herein described. In fact, morphologically the calf’s neoplasm was characterized by a solid growth, without a category-specific histological pattern and the cells were highly atypical, with pleomorphism, severe anisocytosis and anisokaryosis and prominent mitotic activity.

The affected calf reported in this study was seropositive for Schmallenberg virus (SBV) and we speculate that the calf has been infected in utero. SBV is an arthropod-transmitted pathogenic orthobunyavirus of the family *Peribunyaviridae*, of the newly established order *Bunyavirales* that has been associated with congenital malformations of the nervous and musculoskeletal systems and stillbirths in ruminants [14,15]. Recently, a high sequence variability has been noticed in the aminoterminal part of the glycoprotein Gc-encoding region of viruses present in the brain of malformed newborns being potentially involved in immune evasion mechanisms [16]. However, to the best of our knowledge, SBV has not been associated with congenital neoplasms. Further studies would be needed to investigate a possible influence of a trans-uterine infection of SBV and the development of congenital neoplasms.

## 4. Conclusions

We performed a complete clinical and pathological investigation of the crossbred calf revealing a suborbital mass with infiltration of the underlying soft tissues and bone, which was compatible with a congenital form of undifferentiated sarcoma. To the best of our knowledge, our report constitutes the first clinical, ultrasonographic, radiographic, endoscopic and pathological study of a congenital undifferentiated sarcoma, a condition that has been rarely reported in the veterinary literature.

## Data Availability

Not applicable.
